# Supplementary material for: A high-resolution mRNA expression time course of embryonic development in zebrafish
Source: eLife. 2017 Nov 16;6:e30860. doi: 10.7554/eLife.30860 (PMC5690287; doi:10.7554/eLife.30860)
Supplement: Supplementary file 6. [file elife-30860-supp6.zip › biolayout-clusters-files/Cluster139-genes.html]

Cluster139


# Cluster139: Genes

| Ensembl ID | Gene Name | Chr | Start | End | Biotype |
| --- | --- | --- | --- | --- | --- |
| ENSDARG00000090971 | cc2d2a | 23 | 2256141 | 2320942 | protein\_coding |
| ENSDARG00000031293 | dnajc9 | 12 | 9459803 | 9472560 | protein\_coding |
| ENSDARG00000016548 | eif5b | 1 | 45233554 | 45264031 | protein\_coding |
| ENSDARG00000100060 | exosc7 | 19 | 2794248 | 2803285 | protein\_coding |
| ENSDARG00000043665 | glrx5 | 20 | 10496775 | 10500711 | protein\_coding |
| ENSDARG00000045308 | pola1 | 24 | 23695675 | 23787351 | protein\_coding |
| ENSDARG00000003938 | rpa1 | 15 | 26565463 | 26606113 | protein\_coding |
| ENSDARG00000007438 | ube2ib | 24 | 37213010 | 37227532 | protein\_coding |
| ENSDARG00000015998 | wdhd1 | 17 | 15141832 | 15181464 | protein\_coding |
